# Safety and Efficacy of Laparoscopic Caudate Lobectomy: A Systematic Review

**DOI:** 10.3390/jcm10214907

**Published:** 2021-10-24

**Authors:** Panagiotis Dorovinis, Nikolaos Machairas, Stylianos Kykalos, Paraskevas Stamopoulos, Spyridon Vernadakis, Georgios C Sotiropoulos

**Affiliations:** 1 2nd Department of Propaedeutic Surgery, Medical School, National and Kapodistrian University of Athens, 11527 Athens, Greece; kykalos@gmail.com (S.K.); pstamop@gmail.com (P.S.); gsotirop@yahoo.com (G.C.S.); 2 Transplantation Unit, Laiko General Hospital, 11527 Athens, Greece; svernadakis@yahoo.com

**Keywords:** caudate lobe, segment I, spiegel lobe, laparoscopic, liver resection, hepatectomy, morbidity, mortality

## Abstract

Resection of the caudate lobe of the liver is considered a highly challenging type of liver resection due to the region’s intimacy with critical vascular structures and deep anatomic location inside the abdominal cavity. Laparoscopic resection of the caudate lobe is considered one of the most challenging laparoscopic liver procedures. The objective of our systematic review was to evaluate the safety, technical feasibility and main outcomes of laparoscopic caudate lobectomy LCL. A systematic review of the literature was undertaken for studies published until September 2021. A total of 20 studies comprising 221 patients were included. Of these subjects, 36% were women, whereas the vast majority of resections (66%) were performed for malignant tumors. Tumor size varied significantly between 2 and 160 mm in the largest diameter. The mean operative time was 210 min (range 60–740 min), and estimated blood loss was 173.6 mL (range 50–3600 mL). The median hospital length of stay LOS was 6.5 days (range 2–15 days). Seven cases of conversion to open were reported. The vast majority of patients (93.7%) underwent complete resection (R0) of their tumors. Thirty-six out of 221 patients developed postoperative complications, with 5.8% of all patients developing a major complication (Clavien–Dindo classification ≥ III).No perioperative deaths were reported by the included studies. LCL seems to be a safe and feasible alternative to open caudate lobectomy OCL in selected patients when undertaken in high-volume centers by experienced surgeons.

## 1. Introduction

Despite the initial skepticism and reluctance by hepatobiliary surgeons, laparoscopic liver surgery (LLS) is currently considered a safe and efficient mainstream approach for the treatment of benign and malignant liver tumors [1,2]. LLS is considered a valid alternative to the traditional open approach for a plethora of lesions in selected patients [3,4,5,6]. With gradual accrual of experience, certified hepatobiliary surgeons with a strong background in the field of minimally invasive approaches are able to safely and efficiently perform major and complex laparoscopic liver resections [3,7,8,9]. Widely performed complex procedures include resections in posterosuperior segments, combined resections with radical lymph node dissection, repeat laparoscopic liver resections andlaparoscopic liver retrievals for live donor liver transplantation [5,9,10,11,12].

Resection of the caudate lobe of the liver is considered a highly challenging type of liver resection due to the region’s intimacy with critical vascular structures and its deep anatomic location inside the abdominal cavity even via the traditional open approach [13]. Laparoscopic resection of the caudate lobe is likewise considered one of the most challenging procedures through the laparoscopic approach and is part of the “difficult” posterosuperior segments along with segments IVa, VII and VIII [9,14]. Laparoscopic caudate lobectomy (LCL) is only carried out in few experienced centers and only a small number of case reports and case series have been published so far.

To date, no systematic review is available concerning the safety and efficacy of these challenging laparoscopic liver resections. The objective of our systematic review was to evaluate the safety, technical feasibility and main outcomes of LCL.

## 2. Materials and Methods

### 2.1. Sources Search and Selection Criteria

Our study was designed according to the Preferred Reporting Items for Systematic Reviews and Meta-Analyses (PRISMA) guidelines based on the authors’ predetermined eligibility criteria [15]. All appropriate prospective and retrospective studies addressing outcomes of patients with LCL were considered eligible for inclusion in our systematic review. A comprehensive search of the Medline (PubMed) library was undertaken separately by three authors (PD, SK and NM) with the objective of identifying studies that reported results from LCL, published in the English language. The terms utilized included: “laparoscopic”, “laparoscopic liver resection”, “laparoscopic hepatectomy”, “caudate lobe” and “caudate lobectomy”.

Studies reporting at least one postoperative outcome (operative time, estimated blood loss EBL, length of stay LOS, morbidity, and recurrence or survival rates) were included. Exclusion criteria included: (1) animal studies, (2) studies that included patients undergoing procedures other than resection (such as radiofrequency ablation), (3) studies analyzing outcomes after hand-assisted or hybrid techniques and (4) duplicate studies. All articles deemed eligible were subsequently reviewed by all authors and selected for inclusion. The consensus from all authors resolved potential discordances in methodology, selection of articles and statistical analysis.

### 2.2. Data Extraction and Management

Data were extracted from eligible studies and inserted into Excel spreadsheets (Microsoft, Redmond, Washington USA). Data of interest included patient demographics, information on sizes of lesions and perioperative outcomes. Long-term outcomes including overall (OS) and disease-free survival (DFS) were additionally evaluated if available.

## 3. Results

### 3.1. Subsection

**Figure 1:**
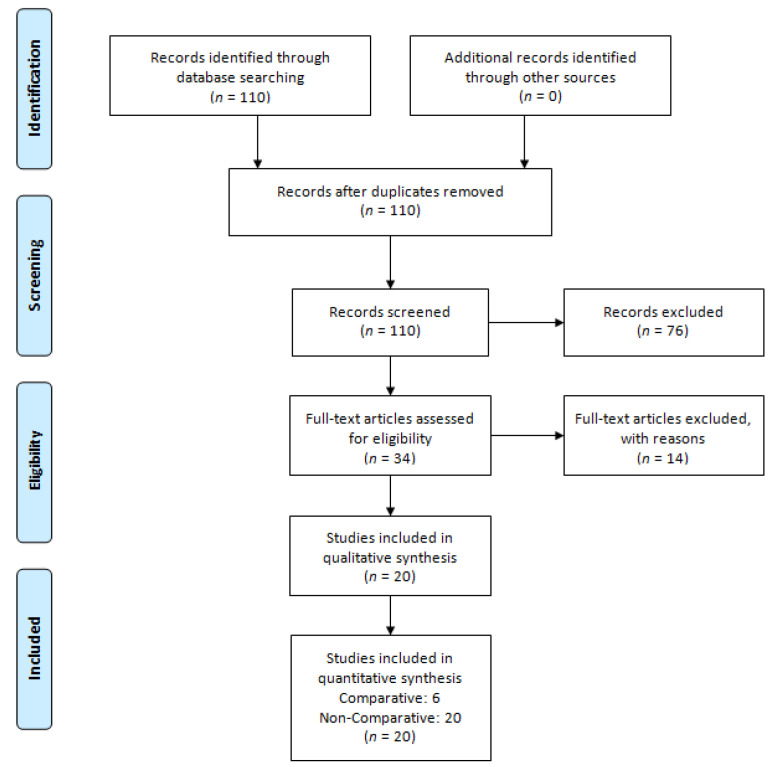
**Prisma** flow diagram for identification of eligible studies.

A total of twenty studies including patients who underwent LCL met our inclusion criteria [16,17,18,19,20,21,22,23,24,25,26,27,28,29,30,31]. Three studies were excluded from our systematic review because they were lacking critical patient and procedure information [14,30,32]. One case report was also excluded because it described partial resection of the inferior vena cava, focusing solely on perioperative data [33]. Molina et al. presented a case report of Spiegel lobe resection in a patient with an accessory left hepatic artery lacking relevant data and was also excluded [34]. One single-center comparative study was also excluded as it did not provide adequate information individually for LCL [35]. Studies involving patients with synchronous resections, commentaries on articles, literature not in theEnglish language and animal studies were also excluded (Figure 1).

Included studies consisted of six single case reports, six case series and five comparative retrospective analyses (four being propensity-score-matched studies) between LCL and open caudate lobectomy (OCL) and three cohort studies. The majority of studies originated from Asia, one from France and one from the US. The indices analyzed were tabulated in the following structured tables: patient characteristics (Table 1) and operative details/short-term outcomes (Table 2). Long-term outcomes (Table 3) were largely unavailable since most of the studies focused on the aspect of technical feasibility, containing data for postoperative morbidity thus having a limited follow-up time.

### 3.2. Main Outcomes

The included studies comprised a total of 221 patients, 36% of whom were women, with ages ranging between 20 and 76 years. The majority of resections, 147 out of 221 cases, representing 63%, were performed for malignant tumors, whereas the remaining 74 cases were carried out for benign pathology. Ishizawa et al. did not specify the lesion pathology of the threereported undertaken resections. Of the resected malignant lesions, 61 were metastatic, the vast majority being colorectal liver metastases, 69 were hepatocellular carcinomas, 3 were intrahepatic cholangiocarcinomas, 2 were perivascular epithelioid cell tumors (PEComas), 1 was aninflammatory pseudotumor-like follicular dendritic cell sarcoma (IPT-like FDC sarcoma), andthe 10 remaining cases were not histologically specified.

Tumor size varied significantly from 2mm to 160 mm in the largest diameter. All included studies reported excision of a single tumor from segment I, while Araki et al. reported simultaneous excision of two tumor foci within the same specimen, and Peng et al.reported two cases of multiple foci within the caudate lobe.

Analysis of perioperative outcomes for patients undergoing LCL revealed a mean operative time of 210 min (range 60–740min). The reported median estimated blood loss (EBL) was 173.6 mL (range 50–3600 mL). Only seven cases of conversion amidst 221 laparoscopic caudate lobe resections were reported, representing 3.1%, while resection margins were found to be positive (R1) in fourteen cases (6.3%). Out of these 14 cases, 10 originate from Capelle et al., where tumors detached from major vessels were considered as R1 vascular [36].

Of 221 patients, 36 developed postoperative complications, 23 of which were minor classified as Clavien–Dindo grade I–II, and 13 were major (CD III or greater) [40]. Minor complications included two cases of postoperative diarrhea, one case of liver failure, two cases of ascites and one of ileus resolving conservatively. Xu et al., Ishizawa et al. and Patrikh et al. each reported a case of bile leakage. Araki et al., aside from the case of ileus CD I-II, reported twomore cases with major complications. More specifically, there was a case of biliary stenosis (CD IIIa) successfully treated by endoscopic stenting and a further one of pancreatic fistula (CD IIIb) resulting in reoperation for the definitive management of this complication. Peng et al. reported a case of liver failure considered to be a major complication. In the case series by Sun et al., fluid collection in the surgical area was the most common complication because of the anterior hepatic transection approach this group undertook to resect lesions originating in the caudate lobe. The median hospital length of stay (LOS) was 6.5 days (range 2–15 days). No case of perioperative death has been reported among the included studies.

### 3.3. Long-Term Outcomes

Though most of the studies reported a limited follow-up time of 90 days postoperatively, focusing solely on postoperative morbidity and not overall survival (OS) and disease-free survival (DFS), Oh et al. reported a median follow up time of 54.6 months, during which 2 out of 4 patients (50%) showed tumor recurrence. Patrikh et al. reported a rate of 42.9% of 5-year disease-free survival and a 76.2% of 5-year overall survival rate. There was one case of lesion recurrence at segment VIII 55 months following LCL, which was treated with radiofrequency ablation (RFA) and a case of carcinomatosis 9 months after the initial resection, treated with adjuvant systemic chemotherapy. The median follow-up time among other studies (Cheung, Wan et al., Dulucq et al., Ho et al., Li et al. and Chen et al.) reached 7.5 months during which no disease recurrence was detected (12, 6, 7, 1, 8 and 13), whereas Patrikh et al. reported the longer follow-up period extending up to 43 months. No deaths were reported in any of the studies in the postoperative observation period (Table 3).

### 3.4. Comparative Studies

Six of the included studies reported comparative outcomes with patients undergoing OCL (Table 4). Three studies reported a statistically significant lower operative time in favor of the laparoscopic approach. All six studies reported a shorter length of stay for patients undergoing LCL compared to the OCL group. Only three studies reported comparison for R0 resection. In the first one (Xu et al.), though in every specimen the malignant lesion was totally removed, 8 out of 18 specimens in the LCL group had a surgical margin <1 cm compared to 28 out of 36 specimens for the OCL group having a surgical margin of <1 cm demonstrating a statistically significant difference between the two groups. This was not confirmed by Patrikh et al., who reported solely R0 resections in the LCL group and 8 out of 9 R0 resections in the OCL group, respectively. Ruzzenente et al. reported 26 out of 30 R0 resections for the LCL group compared to 27 out of 30 R0 resections for the OCL group after the propensity-matching score. Four out of six studies showed similar postoperative complications, while no deaths were reported in any of the studies. Lastly, Ding et al. evaluated hospital costs among the two approaches showing no significant difference.

## 4. Discussion

According tothe cumulative outcomes from our systematic review, LCL is a safe and efficient alternative to open resection for selected patients when performed by experienced hepatobiliary surgeons. This minimally invasive approach combines a low overall morbidity rate of 16.3%, as well as low mortality. Comparative studies also substantiate these outcomes, reporting lower morbidity between laparoscopic and open caudate lobectomy. Moreover, aside from being a safe alternative, is also efficient, with a 98% rate of R0 resections and a 3.1% rate of conversion to open.

Resections of primary and metastatic lesions located within the caudate lobe are considered technically challenging, due to its deep intra-abdominal location and proximity to major vascular structures. The caudate lobe entails three distinct subsegments: (a) Spiegel’s lobe, located behind ligamentum venosum and on the left of the inferior vena cava (IVC); (b) the caudate process, extending to the left and useful in traction of the caudate lobe; and (c) the paracaval portion (Couinaud’s segment IX) corresponding to the dorsally located hepatic tissue in front of the inferior vena cava. Left and right portal veins, along with the hilar bifurcation area, provide the branches supplying the caudate lobe subregions, while short hepatic veins drain blood directly to the IVC [41,42].

Resection of the caudate lobe remains one of the most demanding resections, even through the open approach, while proper management of the short hepatic veins is of cardinal importance. Laparoscopy offers caudal-to-cephalad vision, which results in better exposure and control of the short hepatic veins. Other advantages include proper staging and determination of resectability since tumor seeding, and occult liver and lymph node metastases are identified intraoperatively [43,44]. However, laparoscopy poses a risk of massive intraoperative bleeding occurring from the anterior IVC, which may occur when dealing with tumors larger than 5cm, potentially invading the IVC and found close to the hilum or major hepatic veins [45]. In these situations, obtaining an adequate resection margin, despite laparoscopy’s aforementioned advantages, can be challenging.

**Figure 2:**
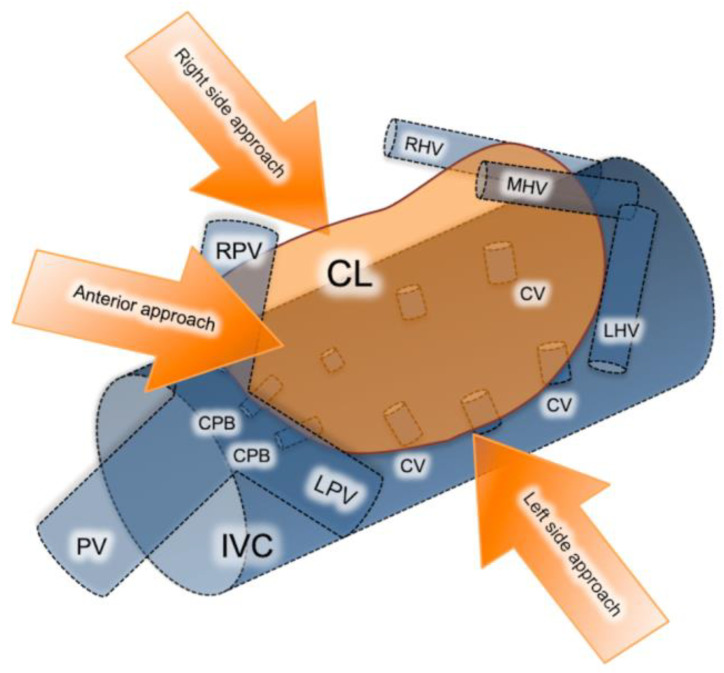
Schematic presentation of the caudate lobe’s anatomic region, blood supply, vascular proximity and main surgical approaches. CL, caudate lobe; CV, caudate vein; LHV, left hepatic vein; MHV, middle hepatic vein; RHV, right hepatic vein; PV, portal vein; LPV, left portal vein; RPV, right portal vein; CPB, caudate portal branch; IVC, inferior vena cava.

There are three different approaches for isolated laparoscopic excision of the caudate lobe described in the literature including the left, right and anterior trans parenchymal, each chosen depending on the tumor size and location (Figure 2) [23]. Decision on which approach is more suitable is made based on tumor localization and size. The right-sided approach is usually reserved for bulky tumors. Taking into consideration that this approach requires a complete rotation of the right lobe to the left in order to expose the caudate hepatic veins, it is quite difficult to perform laparoscopically. The left-sided approach on the other hand is preferred for smaller-size tumors located in the Spiegel process or paracaval portion. Lastly, the anterior trans parenchymal approach includes the performance of retrohepatictransparenchymal resection and suspension of the right lobe [23]. It is furthermore recommended by several authors as a preferred alternative for lesions exceeding 4cm in diameter, or lesions involving critical vascular structures such as the inferior vena cava IVC and short hepatic veins. From a technical standpoint, it prevents hepatic rotation and subsequent venous rupture, making it feasible to dissect the liver parenchyma along the IVC [43].

The conclusions from a meta-analysis by Ding et al., comparing the safety and feasibility of the laparoscopic versus open caudate lobe resection, come in accordance withour results [46]. The included studies were derived predominately from Chinese literature and comprised 237 patients in total. LCL resulted in reduced intraoperative blood loss (*p* < 0.0001), shorter postoperative length of stay (*p* = 0.001), shorter operative time (*p* = 0.0005) and reduced need for intraoperative transfusion. No statistically significant differences in postoperative complications such as bile leaks, ascites and incisional infections were reported. The authors concluded that LCL could be a safe and feasible alternative to open caudate lobectomy OCL, yet lacking solid evidence provided by multicenter randomized control trials and prospective clinical trials [46].

### Methodological Considerations

Several inherent limitations to our study ought to be acknowledged. Outcomes were derived from retrospective, single-center studies with a limited number of operated patients, and as such, selection bias may apply. The included studies reported outcomes for a plethora of benign and malignant tumors; thus, underlying hepatic quality may differ significantly and postoperative outcomes may vary significantly. The included studies reported outcomes for tumors of variable size and extents of resection. For malignant lesions resected, long-term outcomes were not homogeneously reported, and as such, further analysis was not performed. Lastly, variable surgeon and center experience with LCL may impact perioperative outcomes.

## 5. Conclusions

Although all available studies regarding the safety and efficacy of LCL entail various limitations, the reported outcomes remain encouraging. Similarly to other LLS procedures, selection of patients and relevant laparoscopic hepatobiliary surgical expertise remain of great significance and represent decisive factors for the safety and accordingly oncologic efficacy of the procedure. Additional higher-quality studies are warranted to further elucidate whether the laparoscopic approach is beneficial for patients with isolated benign and malignant tumors of the caudate lobe.

## Figures and Tables

**Table 1:** Patient and tumor characteristics.

Author	N	Age, (Years)	BMI (kg/m^2^)	Malignant Tumor				Benign Tumor	Tumor Size (cm)	No. of Lesion	NAT (%)
				Metastatic	HCC	ICC	Other				
Kokkalera et al. [24]	1	37	n/a	0	0	0	0	1	5.2 × 3.5 × 2	1	-
Cheung [18]	1	54	n/a	1	0	0	0	0	2	1	1(100%)
Wan et al. [30]	1	61	n/a	0	0	1	0	0	4.6 × 3.9	1	0(0%)
Dulucq et al. [20]	1	56	n/a	1	0	0	0	0	3	1	1(100%)
Ho et al. [21]	1	61	n/a	0	1	0	0	0	1	1	0(100%)
Oh et al. [27]	4	62(31–71) ^a^	n/a	0	3	1	0	0	1.45 × 1.4 (5 × 3–0.9 × 0.8) ^a^	1	n/a
Li et al. [26]	3	42.6 ± 7.7 ^b^	n/a	0	0	0	3	0	2.2 ± 0.5 ^b^	1	n/a
Chen et al. [17]	7	51(42–75) ^a^	n/a	3	4	0	0	0	3 × 2 × 2 ^a^	1	n/a
Xu et al. [31]	19	47.3 ± 12.7 ^b^	24.3 ± 3.1 ^b^	1	7	0	0	11	3.87 ± 1.1 ^b^	1 ^b^	n/a
Jin et al. [23]	12	50(23–60) ^a^	n/a	0	7	0	0	5	5.2 ± 0.69 ^b^	1	n/a
Araki et al. [16]	15	64 ± 9 ^b^	25.3 ± 4.7 ^b^	12	1	0	0	2	1.95 ± (0.2–5) ^a^	1(1–2) ^a^	3(20%)
Ishizawa et al. [22]	3	n/a	n/a	n/a	n/a	n/a	n/a	n/a	n/a	n/a	n/a
Ding et al. [19]	10	48 ± 17.01 ^b^	n/a	n/a	n/a	n/a	5	5	6 ± 1.41 ^b^ × 5.5 ± 1.91 ^b^	1	n/a
Salloum et al. [29]	5	65.6 ± 18.3 ^b^	n/a	0	4	0	0	1	n/a	n/a	n/a
Kyriakides et al. [25]	1	20	n/a	0	0	0	0	1	6 × 4	1	-
Peng et al. [28]	31	50(24–73) ^a^	21.9(17.2–30.4) ^a^	4	10	0	0	17	4(1–10) ^a^	n/a	n/a
Parikh et al. [37]	12	62(38–89) ^a^	24.56(20.97–30.14) ^a^	1	11	0	0	0	2(0.9–4.1) ^a^	n/a	n/a
Sun et al. [38]	15	43.4 ± 14.2 ^b^	n/a	0	4	0	0	11	6.1 ± 3.4 ^b^	1–2	n/a
Ruzzenente et al. [39]	47	60 ± 15 ^b^	25 ± 25 ^b^	16	17	0	1	13	3.7 ± 2.9 ^b^	n/a	n/a
Capelle et al. [36]	32	61 ± 16 ^b^	n/a	22	0	1	4	5	2.2(0.7–5.8) ^a^	n/a	n/a

Abbreviations: ^a^ median (range); ^b^ mean ± Standard Deviation; n/a, not available; NAT, Neoadjuvant therapy.

**Table 2:** Perioperative outcomes.

Author	Operating Time (min)	Resection Margin (mm)	EBL (mL)	Conversion	Approach Type	Morbidity	Hospital Stay (Days)
Kokkalera et al. [24]	160	n/a	50	No	n/a	0	2
Cheung [18]	180	R0	220	No	Left lateral	0	3
Wan et al. [30]	300	R0	180	No	Caudal	0	7
Dulucq et al. [20]	150	R0	200	No	Left	0	10
Ho et al. [21]	270	R0(3)	200	No	Left lateral	0	4
Oh et al. [27]	241(168–568) ^a^	R0 (0,6) ^a^	180 (120–360) ^a^	No	Left lateral	0	7(6–7) ^a^
Li et al. [26]	225 ± 14.6 ^b^	R0	100(100–200) ^a^	No	2 Left 1 Combined	0	6(6–7) ^a^
Chen et al. [17]	240 ± 7.6 ^b^	R0(8.6 ± 5.4) ^b^	120(10–1000) ^a^	No	n/a	0	6.9(4–11) ^a^
Xu et al. [31]	186.5 (128.5–219) ^a^	R0(<1mm,44%), (>1mm,56%)	75(48.7–200) ^a^	No	Left 14, Right 5	2(11%)	6(4.75–8) ^a^
Jin et al. [23]	140 ± 95.34 ^b^	n/a	57.5(50–350) ^a^	No	n/a	0	8(6–15) ^a^
Araki et al. [16]	150(60–480) ^a^	R0	75(0–500) ^a^	No	Caudate	3(20%)	8 ± 6.5 ^b^
Ishizawa et al. [22]	180–300 ^a^	R0	150–400 ^a^	No	Left	1(33.3%)	7(4–25) ^a^
Ding et al. [19]	216.50 ± 49.59 ^b^	n/a	50(50–125) ^a^	No	n/a	0	15 (11.25–15) ^a^
Salloum et al. [29]	249 ± 65.4 ^b^	R0	280 ± 246 ^b^	No	3 Left, 2 Right	n/a	n/a
Kyriakides et al. [25]	120	n/a	100	No	Left	n/a	n/a
Peng et al. [28]	210(82–495) ^a^	n/a	100(20–1600) ^a^	1	n/a	5(16.1%)	5(2–7) ^a^
Parikh et al. [37]	204.5(75–450) ^a^	0.7(0.1–2.2) ^a^	250(0–650) ^a^	No	Caudate	2(16.7%)	4(2–10) ^a^
Sun et al. [38]	338 ± 124.8 ^b^	R0	706 ± 800 ^b^	No	Anterior	13(87%)	10 ± 3 ^b^
Ruzzenente et al. [39]	309 ± 116 ^b^	R0 29/33	175 ± 153 ^b^	3	n/a	8(17%)	4.9 ± 3.7 ^b^
Capelle et al. [36]	155(29–440) ^a^	R0 22/32	100(50–275) ^a^	3	n/a	2(6.3%)	3(1–39) ^a^

Abbreviations: ^a^ median (range); ^b^ mean ± Standard Deviation; n/a, not available; EBL, Estimated blood loss.

**Table 3:** Long-term outcomes.

Author	Follow-Up (Months)	Adjuvant Chemotherapy (%)	Recurrence, N (%)	Overall Survival (Months)	Disease-Free Survival (Months)
Cheung [18]	12	1 (100%)	No	12	12
Wan et al. [30]	6	1 (100%)	No	6	6
Dulucq et al. [20]	7	1 (100%)	No	7	7
Ho et al. [21]	1	n/a	No	1	1
Oh et al. [27]	54.6 (12.9–86.7) ^a^	1 (25%)	2 (50%)	54.6 (12.9–86.7) ^a^	32 (9–55) ^a^
Li et al. [26]	8 ^a^	n/a	n/a	8 ^a^	8 ^a^
Chen et al. [17]	13 (3–56) ^a^	n/a	n/a	13 (3–56) ^a^	n/a
Kyriakides et al. [25]	n/a	No	No	n/a	n/a
Parikh et al. [37]	43(4–149) ^a^	n/a	n/a	n/a	n/a
Sun et al. [38]	n/a	n/a	13.3%	n/a	n/a
Ruzzenente et al. [39]	n/a	n/a	n/a	n/a	n/a
Capelle et al. [36]	14(10–23) ^a^	n/a	129(54.5%)	85% ^c^	10

Abbreviations: ^a^, median (range); ^c^, 5-year survival; n/a, not available.

**Table 4:** Comparative studies’ outcomes LCL vs. OCL.

Author	N	Age, (Years)	Operative Time (min)	Blood Loss (mL)	Length of Stay (Days)	R0 Resection	Total Complications	Bile Leak
Li et al. [26]	3 vs. 8	42.67 ± 9.45 ^b^ vs. 47.62 ± 8.85 ^b^	225± 18 ^b^ vs. 264± 59 ^b^	133± 33 ^b^ vs. 368± 105 ^b^ *p* = 0.22	6.3± 0.3 ^b^ vs. 15.5± 2.3 ^b^ *p* = 0.006	n/a	1 vs. 3 *p* = 0.72	n/a
Xu et al. [31]	18 vs. 36	48.22 ± 12.59 ^b^ vs. 46.68 ± 11.43 ^b^	186.5(128.5–219) ^a^ vs. 200.0 (163.75–238) ^a^	75(48.75–200) ^a^ vs. 200(100–325) ^a^ *p <* 0.001	6(4.75–8) ^a^ vs. 8(7–9) ^a^ *p* = 0.003	8 vs. 28 *p* = 0.021	2 vs. 4	1 vs. 3 *p* = 0.649
Ding et al. [19]	10 vs. 12	48 ± 17.01 ^b^ vs. 59.22 ± 7.87 ^b^	216.50 ± 49.59 ^b^ vs. 372.78± 96.73 ^b^	50 (50–125) ^a^ vs. 300 (200–350) ^a^ *p* = 0.004	15(11.25–15) ^a^ vs. 16(15–25) ^a^ *p* = 0.034	n/a	0 vs. 5 *p* >0.05	0 vs. 1 *p* = 0.545
Peng et al. [28]	31 vs. 71	50(24–73) ^a^ vs. 48(18–79) ^a^	210(82–495) ^a^ vs. 195(105–375) ^a^	100(20–1600) ^a^ vs. 200(30–1000) ^a^ *p* = 0.017	5(2–7) ^a^ vs. 6(4–39) ^a^ *p <* 0.001	n/a	5 vs. 16 *p* = 0.462	0 vs. 0
Parikh et al. [37]	12 vs. 9	62(38–89) ^a^ vs. 62(48–68) ^a^	204.5(75–450) ^a^ vs. 200(120–550) ^a^	250(0–650) ^a^ vs. 400(100–1500) ^a^	4(2–10) ^a^ vs. 7(2–27) ^a^	12/12 vs. 8/9	2(16.7%) vs. 3(33%)	1 vs. 0
Ruzzenente et al. [39]	47 vs. 177	60± 15 ^b^ vs. 58 ± 14 ^b^	309 ± 116 ^b^ vs. 235± 120 ^b^	175 ± 153 ^b^ vs. 343 ± 292 ^b^	4.9± 3.7 ^b^ vs. 8.7 ± 9.9 ^b^	26/30 vs. 27/30	6 vs. 3	0 vs. 7

Abbreviations: ^a^, median (range); ^b^, mean ± Standard Deviation; n/a, not available; LCL, laparoscopic caudate lobectomy; OCL, open caudate lobectomy.

## Data Availability

Data sharing is not applicable.
